# Administration of electroconvulsive therapy for depression associated with deep brain stimulation in a patient with post-traumatic Parkinson’s Disease: a case study

**DOI:** 10.1186/s12888-016-1108-y

**Published:** 2016-11-15

**Authors:** Miles G. Cunningham, Golnaz Yadollahikhales, Gordana Vitaliano, Craig van Horne

**Affiliations:** 1 Department of Psychiatry, Laboratory for Neural Reconstruction, McLean Hospital, Harvard Medical School, 115 Mill Street, Belmont, MA 02478 USA; 2 Department of Psychiatry, Brain Imaging Nanotechnology Group, McLean Hospital, Harvard Medical School, 115 Mill Street, Belmont, MA 02478 USA; 3 Department of Neurosurgery, Deep Brain Stimulator Center, University of Kentucky, 800 Rose St, Lexington, KY 40538 USA

**Keywords:** Traumatic brain injury, Parkinson’s Disease, Deep brain stimulation, Major depressive disorder, Electroconvulsive therapy

## Abstract

**Background:**

Deep brain stimulation (DBS) has been shown to be effective for parkinsonian symptoms poorly responsive to medications. DBS is typically well-tolerated, as are the maintenance battery changes. Here we describe an adverse event during a battery replacement procedure that caused rapid onset of severe depression.

**Case Presentation:**

The patient is a 58-year-old woman who was in a serious motor vehicle accident and sustained a concussion with loss of consciousness. Within weeks of the accident she began developing parkinsonian symptoms that progressively worsened over the subsequent 10 years. Responding poorly to medications, she received DBS, which controlled her movement symptoms. Five years after initiating DBS, during a routine battery change, an apparent electrical event occurred that triggered the rapid onset of severe depression. Anti-seizure and antidepressant medications were ineffective, and the patient was offered a course of electroconvulsive therapy (ECT), which resulted in complete reversal of her depressive episode.

**Conclusion:**

Parkinson’s syndrome can be seen after a single closed head injury event. Post-traumatic parkinsonism is responsive to DBS; however, DBS has been associated with an infrequent occurrence of dramatic disruption in mood. ECT is a therapeutic option for patients who develop intractable depressive illness associated with DBS.

## Background

While multiple events of head trauma are known to increase the risk of a Parkinson’s syndrome and parkinsonism can be seen in patients with chronic traumatic encephalopathy (CTE) [1], the development of Parkinson’s syndrome after a single event of head trauma has been infrequently reported [2]. Criteria for post-traumatic Parkinson’s syndrome are that i) the head trauma should result in concussion or unconsciousness, ii) parkinsonian features must appear soon after the traumatic event, and iii) the course of the subsequent Parkinson’s syndrome should be uninterrupted [2]. An additional criterion is evidence that there occurred structural damage to the midbrain [3]; however, this may not be evident with neuroimaging and may only be seen in post-mortem studies. Trauma-induced parkinsonism is associated with damage to the nigrostriatal pathway caused by disruption of the blood-brain barrier, edema, leukocyte infiltration, microglial activation, and free radical production [4]. Furthermore, impaired axonal transport or axonal sheer stress, especially in the substantia nigra, can result in the accumulation of α-synuclein [5]. In addition, genetic predisposition to Parkinson’s disease (PD) may increase the susceptibility to parkinsonism after head trauma [6]. Although treatment options are essentially the same as for those suffering from idiopathic PD, patients with post-traumatic Parkinson’s syndrome are often refractory to conventional pharmacologic treatment [2, 7]. Alternative treatment options do exist, such as deep brain stimulation (DBS), which has been established as an effective treatment option for PD as well as other movement disorders. DBS therapy, however, carries with it various risks. In addition to the possible surgical complications and the sensory and motor side effects, dramatic mood disturbances have been reported, notably depression with suicidal ideation. In such cases where standard medication and psychotherapy have failed, electroconvulsive therapy (ECT) can be safely administered without adverse events [8, 9], and concerns such as displacement or increasing the temperature of the stimulating electrode(s) have been assuaged [10, 11]. Moreover, ECT has been shown to reduce movement dysfunction in PD, independent of its effects on mood [12], presumably by increasing dopaminergic transmission in the striatum [13]. This manuscript is in compliance with the CARE guidelines for reporting case studies.

## Case Presentation

The patient is a 58-year-old woman who 18 years prior (1998) was the driver in a severe motor vehicle accident (MVA) during a routine work commute in which her vehicle flipped end-over-end at interstate speed. She lost consciousness for several minutes and experienced cervical and upper thoracic pain, but otherwise appeared uninjured. Imaging studies of her head and spine were unremarkable, and she was discharged the same day with a cervical collar. Four months later, she began to experience tremor in her left hand and was seen by a neurologist. Repeat magnetic resonance imaging (MRI) showed small hyperintensities in the left temporal and anterior frontal lobes, hippocampus, amygdala, and putamen, which were thought to be posttraumatic. Over the ensuing months, she developed more dramatic left upper extremity tremor and left-sided bradykinesia with decreased arm swing and shortened left stride. She then developed right upper extremity involvement and bilateral cogwheeling, masked faces, hypophonia, unstable, shuffling gait, and freezing. With the development of these symptoms, the patient reported episodes of anxiety and depression as well as occasional passive suicidality. She also experienced rigidity and pain in her neck and upper back and found it difficult to catch her breath. This exacerbated her anxiety, and she became preoccupied that she might lose her ability to breath and she became terrified of being alone. Prior to the accident, the patient had no psychiatric or medical history. Her family history was also unremarkable. She did not smoke, and while she was a mild social drinker, she had no experiences with drugs of abuse. Lumbar puncture, heavy metal screening and serum ceruloplasmin tests were negative. As her symptoms were not consistent with idiopathic PD, she was diagnosed with post-traumatic Parkinson’s syndrome.

The patient was given trials of antiparkinson agents, but found the side effects intolerable. Modest effects were seen with a low dosage of carbidopa/levodopa, but the patient reported that this and other medications often made her symptoms worse. Moreover, she demonstrated a poor response to non-pharmacological interventions such as homeopathy, meditation, herbal medicine and physiotherapy. Four years after the accident, neuropsychological evaluation demonstrated reduced encoding of non-verbal material as well as reduced motor speed and dexterity. In addition, she lost details on copying and had lower than average cognitive proficiency. However, testing revealed no abnormalities in verbal information retention, verbal fluency, language and academic skills, attention, recall, reasoning, and executive functioning.

In 2005 (7 years after her accident) the patient was evaluated for DBS and stimulating electrodes were placed within the subthalamic nuclei bilaterally. Her implantation surgery was performed awake with microelectrode recording, test stimulation, and CRW stereotactic frame guidance. She tolerated the procedure without any perioperative complications or adverse events. Her PD symptoms improved substantially following the initiation of therapeutic stimulation. She was able to return to a nearly independent level of functioning. Her first battery change was in 2008 without incident, and DBS continued to be effective in treating her parkinsonian symptoms. Her mood remained stable, and her anxiety was better controlled, particularly with the relief she experienced in her rigidity.

In March, 2010, her Kinetra pulse generator was replaced with a PC unit to allow for more programming options. While battery replacements are typically performed with local anesthesia and conscious sedation, the patient requested that minimal sedating medication be used. During the battery replacement procedure, the patient reported a rapid onset of uncomfortable tingling sensations and anxiety upon placement of the battery within the surgical pocket. The pulse generator was immediately retrieved, turned off, and re-implanted once the patient’s discomfort resolved. The therapy was re-initiated in the operating room without incident. Given the concern over the event, she was observed overnight as an inpatient. The following day, she reported experiencing numerous brief “surges” followed by periods of anxiety and panic. No loss of consciousness or change in mental status were associated with these events, and no neurologic signs were noted. She was then discharged home with her husband. After recovery from surgery, the patient was left with ongoing episodes of anxiety and within 24 h she developed severe depressive symptoms including sadness, agitation, hopelessness, an impending sense of doom, and morbid suicidal thoughts. While anxiety and depressed mood predominated, the patient reported experiencing brief episodes of “giddiness” every 1-3 days.

**Fig. 1:**
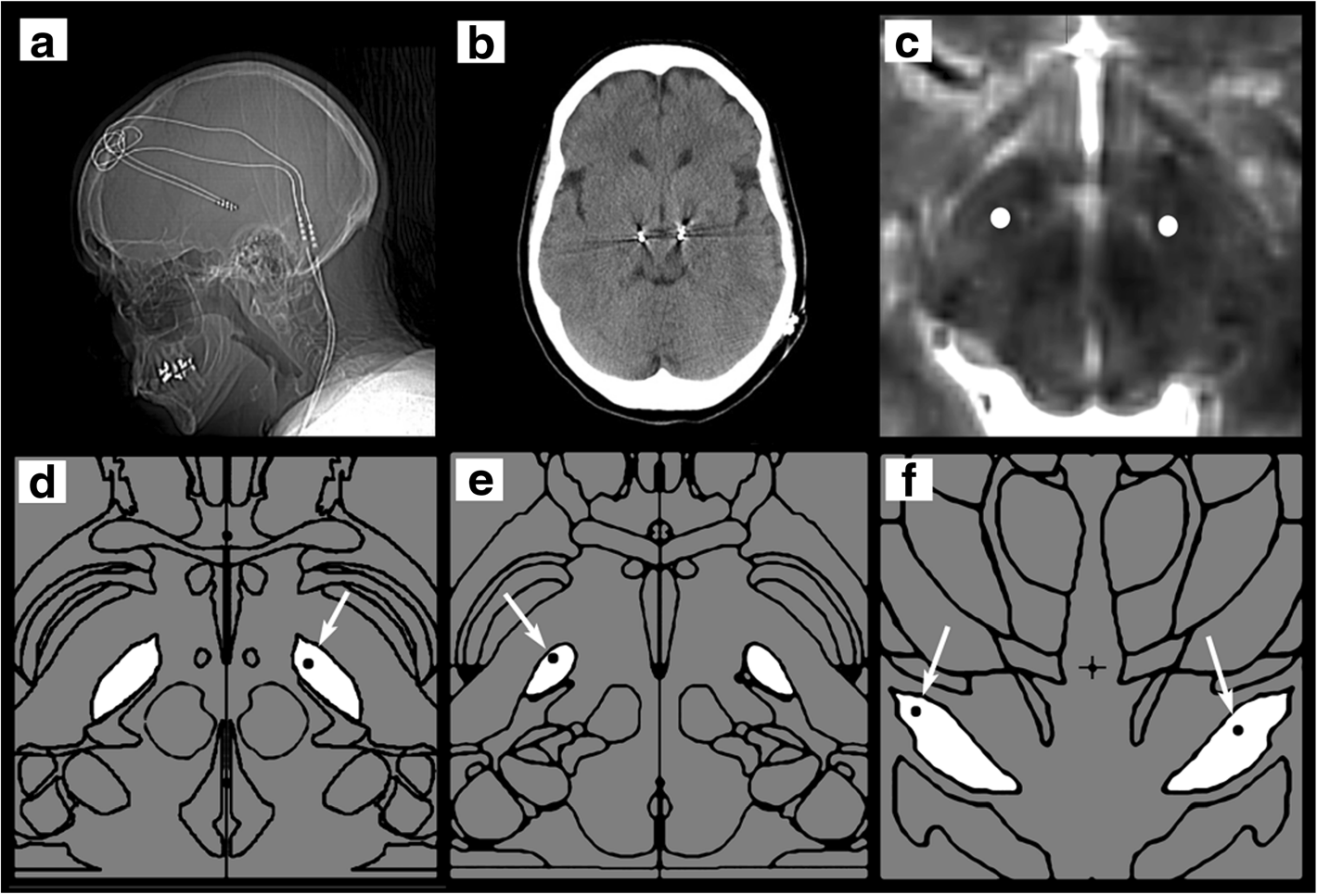
Illustration of ventral-most active lead location within the subthalamic nucleus (STN). Images were created by fusing the pre-operative MRI with post-operative CT images using BrainLab. The deepest active contacts were marked and identified as white dots (**c**) in the axial MRI section through the midbrain at the level of the STN and red nuclei. These illustrate the relative position in the mediolateral and anteroposterior directions. **d**, **e**, and **f** demonstrate the deepest active contacts (white arrows pointing to black dots) within the STN by mapping the contacts into the representative slices from the Schaltenbrand and Wahren Atlas within BrainLab. **a** (axial view), left contact within anteromedial STN. **b** (axial view), right lead within dorsolateral STN and having a more dorsal position compared to (**a**). **c** (coronal view), both active contacts positioned within the dorsal STN

Multiple programming sessions failed to impact her condition and there was concern that the pulse generator was malfunctioning. However, a repeat scan revealed that her intracranial leads had not shifted, and they were indeed appropriately placed (Fig. 1). Lead integrity was evaluated through x-ray imaging and electrical impedance testing of all possible combinations, and there was no evidence of abnormalities. After eliminating all other possible explanations, the patient was brought back to the operating room and her PC pulse generator was replaced by a new Kinetra. Her depression and anxiety persisted, however, and she reported having intrusive thoughts, insomnia, anorexia, and constant feelings of dread. Five days later, overwhelmed with a sense of hopelessness, the patient attempted suicide by overdosing on diazepam and zolpidem. After stabilization as an inpatient, she began receiving care from a neuropsychiatrist to address her psychiatric symptoms in the context of her neurological disease. The patient was exquisitely sensitive to medications and only very low doses were tolerated. She was given unsuccessful trials of benzodiazepines, antidepressants, mood stabilizers, and antipsychotics. After a failed trial of valproate, the patient showed some improvement with lamotrigine. Escitalopram was then added with equivocal success. The patient showed only modest improvement after 1 year of medication trials, psychotherapy, and cognitive behavioral therapy (CBT), as well as alternative medicine interventions, including acupuncture and therapeutically-induced dissociation (TID, Cunningham, unpublished). With her then existing battery losing its charge, and with persisting suspicion of battery malfunction, in 2011, her Kinetra battery was replaced again. Although her parkinsonian symptoms were adequately treated, her severe mood disorder was unrelenting after ongoing medication revisions with continued psychotherapy and CBT. Her stimulation parameters were adjusted numerous times in an attempt to attenuate the mood symptoms while maintaining control over her movement disorder. Neither these adjustments nor turning off the stimulation entirely, had any beneficial effect on her anxiety and depression.

**Fig. 2:**
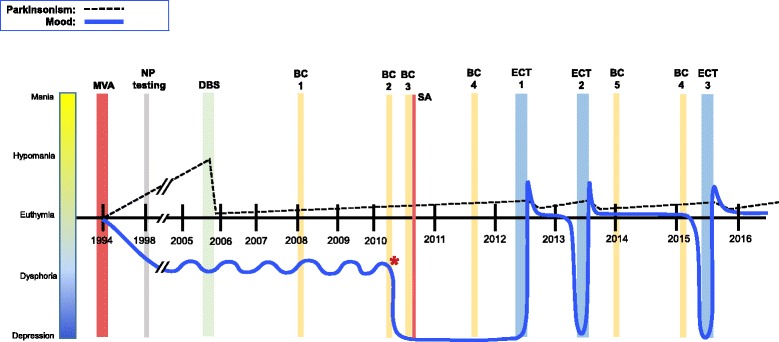
Course of illness depicting parkinsonism, mood, DBS intervention, and ECT treatments. Asterisk marks electrical event followed by immediate and dramatic onset of depression. Note spike in mood toward hypomania with ECT treatments followed by stable euthymic state. Note also transient improvements in parkinsonism after ECT. MVA, motor vehicle accident; NP testing, neuropsychological testing; DBS, deep brain stimulation; BC, battery change; SA, suicide attempt; ECT, electroconvulsive therapy

The patient was then educated on the efficacy of ECT for DBS patients with depression [14]. While initially resistant and fearful, she reconsidered and agreed to proceed with ECT. She underwent 8 bilateral ECT treatments, three times per week. For each treatment, her DBS was interrupted and the stimulator voltage was set to “0” as previously described [14]. The stimulus was given with a MECTA machine at 0.8 mA, PW 1 ms, frequency 40 Hz, and durations from 3 s to 4 s. In the recovery room, the stimulator parameters were returned to their original settings (left: 2.9 V, PW 60, 130 Hz-; right: 2.5 V, PW 60, 130 Hz). After the third ECT treatment, the patient began to show a reduction in anxiety and depression. However, after her forth ECT treatment, she experienced mild short-term memory problems and the duration of ECT was decreased from 4 to 3 s. Over the course of ECT and 1 week subsequent to treatment, she demonstrated complete resolution of depression and experienced minimal anxiety. She also experienced a modest improvement in her parkinsonism, notably feeling nimbler with a more fluid gate. Her Quick Inventory of Depressive Symptomatology scores dropped from 11 to 4 at the end of her ECT treatment course. Her parkinsonism continued to be controlled and she remained euthymic without medications for 10 months. Progressively however, she began to experience sleep disturbance, worsening anxiety, onset of depression and intrusive thoughts, and vague suicidal ideation. Still intolerant of medications, she underwent another course of ECT following a similar procedure as her first course. She received 8 bilateral ECT treatments, but with a slightly modified bifrontal placement of electrodes (0.8 mA, PW 1 ms, frequency 40 Hz, and duration 3 s). After each ECT treatment the stimulator parameters were reset to their original settings (left: 2.5 V, PW 90, 190 Hz-; right: 2.3 V, PW 90, 190 Hz). Again, the patient experienced a complete resolution of depression and anxiety with notable improvement in her parkinsonism lasting approximately 3 months. Her Quick Inventory of Depressive Symptomatology scores dropped from 17 to 4 at the end of this ECT treatment course. She has since demonstrated a near-yearly cycling into depression after a course of ECT. Having now received three courses, the patient is being placed on a maintenance ECT schedule to prevent relapse. See Fig. 2 for patient’s course of events.

## Discussion

PD as a result of a single episode of traumatic brain injury has been a center of debate. Some researchers believe that only severe head trauma resulting in a GCS of <8 can be linked to the development of PD [15]. However, in this case report, the patient had few symptoms and a relatively brief loss of consciousness. Considering her negative family history and her neuropsychological results, along with her rapid onset of symptoms after the trauma, idiopathic PD seemed to be unlikely. She has also met the proposed criteria of post-traumatic PD [3]. Although DBS has been used extensively in patients with idiopathic PD, its efficacy in the setting of post-traumatic PD is unclear. With our patient’s intolerance of pharmacologic interventions, she elected for DBS and demonstrated good clinical results without significant side effects.

DBS has been shown to trigger depressive symptoms and it may worsen a preexisting depressive disorder [7, 16]. Our patient’s profound mood disorder, however, was not seen with the original placement of DBS electrodes nor the delivery of current. Indeed, she responded positively to DBS for approximately 5 years and remained stable. Her rapid and severe worsening of depression occurred during the replacement of a pulse generator. The event and the mechanism that caused the patient’s dramatic mood disturbance are poorly understood. A battery defect was taken into consideration; however, replacement of the battery associated with the adverse event (PC) with the original pulse generator model (Kinetra) did not relieve the patient’s depressive symptoms. We therefore speculate that the event was associated with a sudden onset of stimulation during the battery change. Per our routine, new pulse generators are programmed in the operating room, prior to implantation, to the same therapy settings as the pulse generator being replaced. They are then turned off prior to the procedure, and stimulation is then progressively “ramped up” over approximately 8 min after implantation. This delay feature is built into the pulse generators to allow smooth transitions during stimulation adjustments. In this patient’s case, one of her settings was programmed in the “unipolar” mode, which used the pulse generator as the cathode portion of the circuit, and the battery had not been turned off prior to implantation. Therefore the therapeutic circuit was activated instantaneously, without delay, upon contact of the pulse generator with the patient’s tissue, and the full therapeutic stimulation was delivered without any buffering ramp-up delay.

Electrical events, such as the one that occurred in the present case, are known to result in structural and functional changes resulting in sensitization of neural circuitry. Kindling, for example, is seen experimentally when repeated, or in some instances *single*, subconvulsant currents trigger seizure activity [17]. The kindling phenomenon has been offered as an explanation for the progressive worsening of mood disorders with repeated psychosocial stressors or mood episodes [18, 19]. Moreover, it has been proposed that because the subthalamic nucleus includes a limbic subdivision, which processes emotional and motivational information, unregulated or over-stimulation of this area may be associated with the development of depressive symptoms [20]. It is therefore conceivable that a single or a brief series of stimulations that may have been delivered to our patient rendered her susceptible to depression and anxiety, which was refractory to antidepressant and anti-seizure medications. Fortunately, ECT resulted in complete resolution of her debilitating and life-threatening depressive symptoms.

While the mechanism for DBS-induced depression remains unknown, there may occur a propagation of stimulation from the subthalamic nucleus to the substantia nigra. Taking this mechanism in to account, however, it might be expected that discontinuing stimulation would alleviate the depressive symptoms [16]. Bejjani et al. [16] have reported a case of a 65 year-old woman with PD for whom placing a DBS electrode in the left substantia nigra, 2 mm below the site where stimulation alleviated the signs of Parkinson’s disease caused transient acute depression. The manifestations were resolved within 90 s after stopping the stimulation. However, Chou et al. [7] found that turning off DBS in a patient suffering from psychotic depressive episodes following DBS did not lead to improvements in symptoms. There are other cases in which patients relapsed into depression after DBS placement while having prior history of major depression. This raises the possibility of whether DBS has the ability to trigger depression in patients having an underlying predisposition [7, 14].

## Conclusions

This case report describes a patient with an atypical onset of what is believed to be post-traumatic Parkinson’s syndrome. DBS proved beneficial for her worsening movement disorder similar to its established efficacy for idiopathic PD. While DBS has been linked to the onset of depressive syndromes, these cases are typically gradual in onset. In the present case, however, there occurred a rapid manifestation of severe and intractable depression and anxiety soon after an untoward incident during replacement of a pulse generator. We hypothesize that the patient’s syndrome was associated with an electrical event that permanently rendered her more susceptible to mood disturbances. Complicating her neuropsychiatric syndrome were intolerable side effects of virtually all medications. Fortunately, with a multidisciplinary approach, DBS remained effective for her movement disorder, and she has responded robustly to ECT combined with non-pharmacologic interventions.

## Abbreviations

BC: Battery change
CBT: Cognitive-behavioral therapy
CRW: Cosman- Roberts-Wells
CTE: Chronic traumatic encephalopathy
DBS: Deep brain stimulation
ECT: Electroconvulsive therapy
GCS: Glasgow Coma Scale
MRI: Magnetic Resonance Imaging
MVA: Motor vehicle accident
PD: Parkinson’s Disease
PW: Pulse width
SA: Suicide attempt
STN: Subthalamic nucleus
TID: Therapeutically-induced dissociation
